# The concealed side of caspases: beyond a killer of cells

**DOI:** 10.1007/s00018-024-05495-7

**Published:** 2024-12-03

**Authors:** Lina Abdelghany, Chanin Sillapachaiyaporn, Boris Zhivotovsky

**Affiliations:** 1 Institute of Environmental Medicine, Karolinska Institutet, Stockholm, SE-171 77 Sweden; 2 Engelhardt Institute of Molecular Biology, RAS, Moscow, 119991 Russia; 3 Faculty of Medicine, Lomonosov Moscow State University, Moscow, 119192 Russia; 4 Present Address: Department of Pharmacology and Toxicology, Faculty of Pharmacy, Tanta University, Tanta, 31527 Egypt

**Keywords:** Caspase, Cell death, Survival, Differentiation, Proliferation, Cancer, Inflammation, Neurodegenerative disease

## Abstract

Since the late 20th century, researchers have known that caspases are a pillar of cell death, particularly apoptosis. However, recent advances in cell biology have unraveled the multiple roles of caspases. These enzymes have an unconventional role in cell proliferation, differentiation, and invasion. As a result, caspase deregulation can fuel the fire of cancer, incite flames of inflammation, flare neurodegenerative disorders, and exacerbate skin pathologies. Several therapeutic approaches toward caspase inhibition have been investigated, but can caspase inhibitors harness the maladaptive effect of these proteases without causing significant side effects? A few studies have exploited caspase induction for cancer or adoptive cell therapies. Here, we provide a compelling picture of caspases, starting with their evolution, their polytomous roles beyond cell death, the flaws of their deregulation, and the merits of targeting them for therapeutic implications. Furthermore, we provide a deeper understanding of the evolution of caspase-related research up to the current era, pinpointing the role of caspases in cell survival and aiding in the development of effective caspase-targeted therapies.

**Graphical Abstract:**

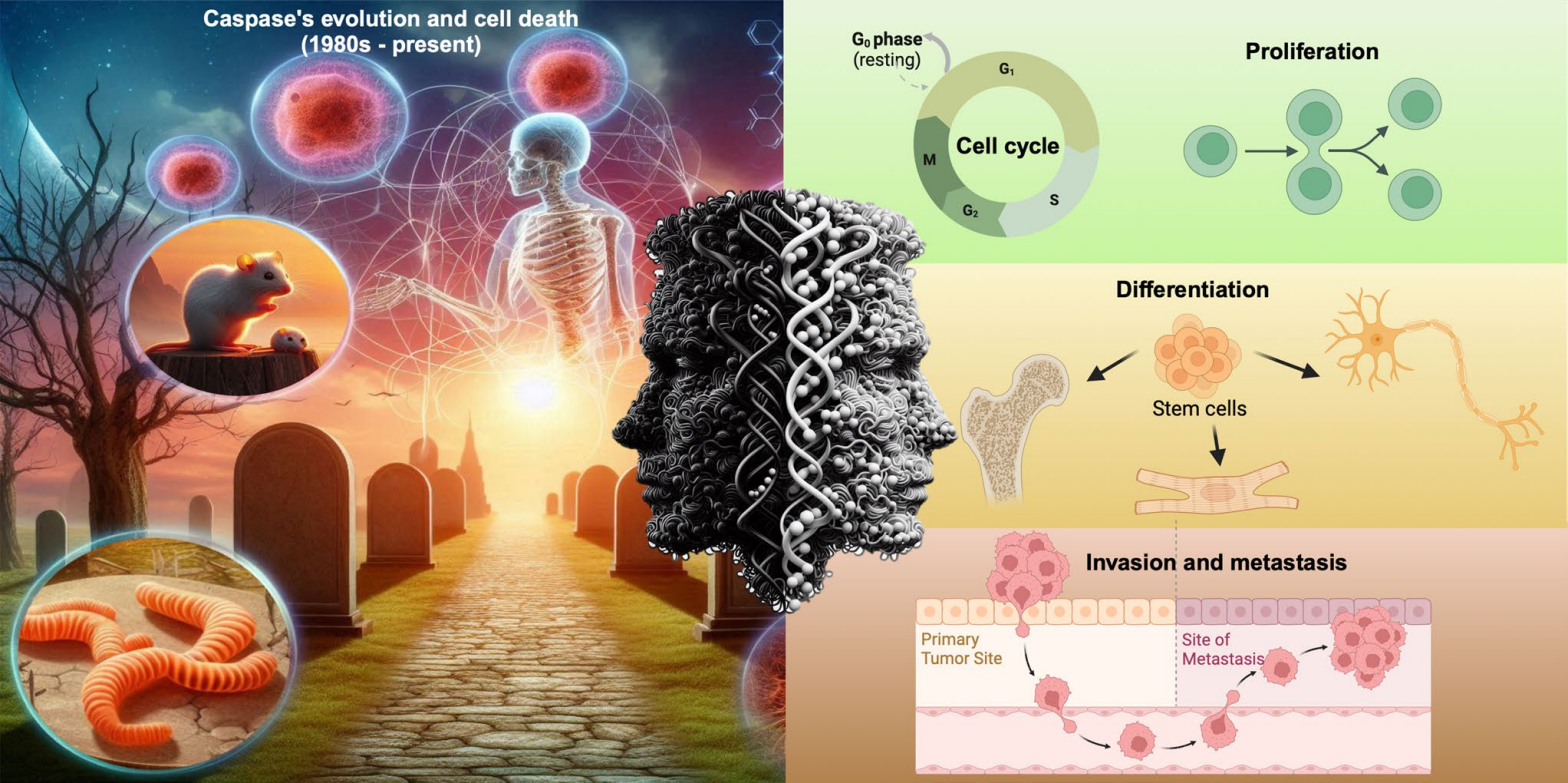

## Introduction

Caspases (cysteine-dependent aspartyl-specific proteases) were originally discovered as an essential enzyme for the maintenance of cell death homeostasis. Caspases exist in all tissues of the body, from the skin to the brain, and their genes are found in a wide range of living organisms, from worms to humans. Nevertheless, does caspase activation always sentence a cell to death? The answer is no: Caspases are not only drivers of cell death, but there is also compelling evidence that they play a role in cell survival [1]. Importantly, certain cells displaying caspase activation also exhibit increased proliferation. This indicates that the cell has the ability to survive the destructive activity of caspases, which can be accomplished in many ways. One is if the degree of caspase activation remains below a certain limit, then the cell might be able to endure and maintain low levels of caspase activity without undergoing cell death. Furthermore, the appropriate expression of specific physiological caspase inhibitors (e.g., inhibitors of apoptosis proteins, IAPs, or Bcl-2 family members) can effectively control caspases and restrict the level of their activation. Evidence demonstrates that cells can reverse apoptosis, even in advanced stages of the process. This phenomenon is referred to as “anastasis”, which translates to “rising to life” in Greek [2].

Caspases have also been demonstrated to contribute to the process of differentiation. Research indicates that caspases play a crucial role in the development of brain and glial cells, osteoblasts, skeletal myoblasts, and several cell types in the hematological system, such as erythrocytes. Caspases additionally show “noncanonical” functions in regulating the cell cycle. Several in vitro and in vivo studies have provided evidence for the potential involvement of caspases in the regulation of the cell cycle in both a positive and negative manner [3]. This indicates that caspases have more intriguing roles in the cells other than apoptosis. A large amount of evidence suggests that dysregulated caspase activation underlies numerous diseases and disorders, including cancer, neurological diseases, psoriasis, and many others. As a result, therapeutic targeting of caspases, either inhibitors or activators, is of great interest as a therapeutic intervention. However, only a limited number of caspase inhibitors have advanced to clinical trials due to the wide range of non-apoptotic functions of these proteins in normal conditions, as well as the persistent challenges with their usage in treating diverse diseases. These limitations include a lack of precise targeting, poor effectiveness, and an increased risk of general toxicity.

The non-apoptotic roles of caspases have been extensively reviewed although not as much as the apoptotic ones [4–7]. Here, we provide an insightful analysis of the various non-cell death functions of these proteins and the complex consequences of their dysregulation. Additionally, we highlight the crosstalk between caspases and diseases such as tumorigenesis and neurodegeneration and the emerging studies on the therapeutic uses of induced caspases to shed light on their enigmatic function and aid in a better understanding of their role. This information can provide better strategies for the development of therapeutic caspase-targeted therapy.

## The evolutionary journey of caspases: from nematodes to Homo sapiens

Caspases belong to the clan CD peptidases of the phylogenetic kingdom and more specifically represent subfamily C14A. These peptidases are known to mediate proteolytic events indispensable for biological phenomena such as cell death and inflammation. Caspases are a unique family of proteases that can cleave proteins on the C-terminal side of aspartate. In addition to caspases, only granzyme B has a similar ability. At the end of the 1980s, researchers identified a novel mammalian protease, interleukin-1b-converting enzyme (ICE), with this proteolytic activity and showed that it plays an important role in the development of inflammation [8]. However, only in 1993 was the homolog of ICE discovered by Robert Horvitz’s group in the nematode *Caenorhabditis elegans* (*C. elegans*) as the first cell death-associated caspase (Ced-3). During the development of *C. elegans*, 131 neuronal cells undergo programmed cell death (PCD). There are four death-related genes (*egl-1*, *ced-9*, *ced-3*, and *ced-4*) that play essential roles in either the initiation or execution of the cell death program in the nematode [9]. Later, researchers found that the *C. elegans* genome has four genes (ced-3, csp-1, csp-2, and csp-3) encoding caspase-like proteins. Among these four proteins, Ced-3 is the most recognized cell-killing enzyme [10]. Caspases are thought to play a pivotal role in apoptosis, the most investigated and best-known mode of PCD, as evolutionarily conserved proteases; however, the number of caspases that have been identified is distinct for each species. This indicates that species-specific functions or diversification of physiological roles have been cultivated through caspase evolution [11].

Since 1993, a family of related proteases has been identified from sponges to vertebrates. In mammals, at least 16 caspase genes have been discovered so far. Caspases in mammals are categorized depending on their function, structure, or cleavage specificity [12]. These proteases are expressed even in insects. The first insect caspase, *Sf caspase-1*, was cloned from *Spodoptera frugiperda*. In 1997, the first *Drosophila melanogaster* caspase (Drosophila caspase-1, DCP-1) was identified, which is structurally similar to Ced-3 [13]. To date, seven caspases have been characterized in insects: three initiators and four effectors [14]. Many studies have shown that caspases are essential for PCD in the fly and are likely regulated like their mammalian counterparts. In mosquitoes, several putative caspases have been identified in the genomes of *Aedes aegypti* and *Anopheles gambiae*. A small number of caspases have been found in *Lepidoptera*, the flour beetle, *Tribolium castaneum*, and the pea aphid, *Acyrthosiphon pisum* [15]. A short form of sponge caspase shows the highest sequence similarity to human caspase-3 and has caspase-3-like enzymatic activity [11]. Zebrafish carry 19 caspase genes, including known orthologs of human caspases [16]. Importantly, zebrafish caspases have distinct expression patterns during development, suggesting both specific and conserved functions among vertebrates (Table 1).

**Table 1:** Overview of caspase gene evolution among species

Organism	Caspase members	Ref.
*Caenorhabditis elegans*	Ced-3, csp-1, csp-2, and csp-3	[21, 23]
*Drosophila melanogaster*	Class I: DREDD, DRONC and STRICA	[14]
	Class II: DCP-1, DRICE, DECAY and DAMM	
Zebrafish	Inflammatory caspases: caspases 1, 19a, 19b, 23	[16]
	Initiator caspases: caspases 2, 9, 8a, 8b, 10, 20, 22	
	Executioner caspases: caspases 3a, 3b, 6a, 6b, 6c, 7, 21, 17	
Human	Inflammatory caspases: Caspases-1, -4, -5 and − 12	[8, 12, 24, 25]
	Initiator caspases: Caspases-2, -8, -9 and − 10	
	Executioner caspases: Caspases-3, -6 and − 7	
	Caspase-14	
	Caspase- 16	
Rodent	Inflammatory caspases: Caspases-1, -11 and − 12	
	Executioner caspases: Caspases-3, -6 and − 7	
	Caspase-14	
Bovine	Caspase-13, Caspase-15	
Opossum	Caspase-16, Caspase-18	

Interestingly, in higher plants genetically controlled mechanisms leading to the selective death of individual cells also involve the regulated interplay of various types of proteases. Some of these enzymes are structurally homologous to caspases and have therefore been termed metacaspases and paracaspases [17]. Additionally, YCA1 is a metacaspase that has an important biological function in the regulation of cell death in yeast [18, 19]. However, it should be emphasized that caspases, metacaspases, and paracaspases are considered to be three separate groups [20].

As described earlier, caspases are present in various eukaryotic organisms and involved in various processes such as cell death, inflammation, and in some cases spermatogenesis and embryogenesis. This raises two main questions: It has been suggested that eukaryotic caspase evolution may stem from the presence of metacaspases within α-proteobacteria, which are hypothesized to be predecessors of the eukaryotic mitochondrion [21]. Given the accumulating evidence that caspase deficiency has a pro-survival effect, another question is does caspase deficiency affect the viability of living organisms, and if so, to what extent? In mice, caspase-8 and caspase-7 knockout results in embryonic lethality. On the contrary, mice that lose caspase-1, caspase-2, caspase-3, caspase-6, caspase-9, caspase-11, or caspase-12 remain viable, although for a significantly different time [22]. Therefore, it is necessary to pay particular attention to caspase deficiency in humans.

## The role of caspases in programmed cell death

**Fig. 1:**
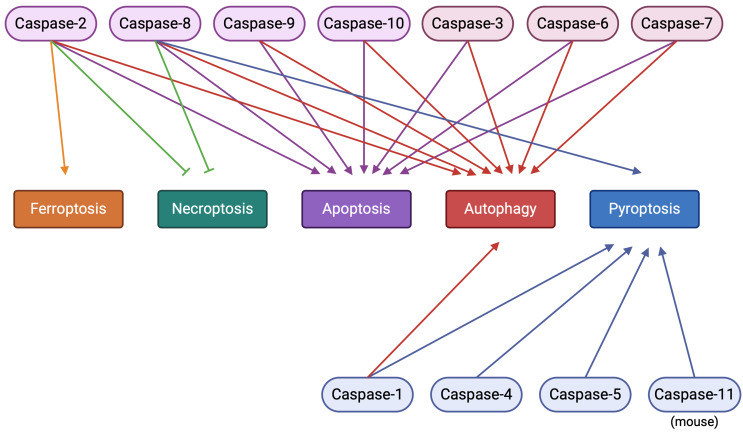
The role of caspases in programmed cell death

Caspases can be classified based on their known functions into two main categories: apoptotic and inflammatory caspases. Apoptotic caspases mediate the signal transduction of apoptotic cell death. These enzymes can be grouped into initiator caspases (caspase-2, caspase-8, caspase-9, and caspase-10) and executioner caspases (caspase-3, caspase-6, and caspase-7). Initiator caspases, which contain the death effector domain (DED, in caspase-8 and caspase-10) or the caspase activation and recruitment domain (CARD, in caspase-2 and caspase-9), are activated during the initial steps of the apoptotic signaling pathway through extrinsic (death receptor) or intrinsic (mitochondrial) signals, respectively. The activation of the initiator caspases will recruit an assembly of multiple proteins to form multi-component complexes and subsequently activate the executioner caspases, leading to the cleavage of cellular proteins and eventually cell death. Inflammatory caspases (caspase-1, caspase-4, and caspase-5 in humans, and caspase-11 in mice) play key roles in the innate immune response against pathogen infection and regulate the production of the pro-inflammatory cytokines: interleukin 1 beta (IL-1β) and IL-18, leading to pyroptosis [8, 26]. In addition, caspases, although not directly, are involved in other forms of PCD including necroptosis, ferroptosis, and autophagy. Inactivation of caspase-8 activity induces receptor-interacting serine/threonine-protein kinase 1 (RIPK1) and RIPK3 then phosphorylates the mixed linage kinase domain-like pseudokinase (MLKL) to form pores on the cell membrane, resulting in necroptosis [27]. In the case of autophagy, caspases can interact directly with autophagy-related proteins (ATGs) and up/downregulate autophagy depending on the cellular protein interaction [28]. Recently, it was shown that caspase-2 might regulate ferroptosis via non-proteolytic interaction with various proteins [29]. Moreover, crosstalk between multiple types of PCD pathways under caspase activation has been reported. For example, caspase-8 is the molecular switch for apoptosis, necroptosis, and pyroptosis: It helps prevent tissue damage during embryonic development and adulthood [30]. Regarding caspase-2, both its positive and negative involvement in the regulation of apoptosis, necroptosis, and ferroptosis have been described [29, 31–33] (Fig. 1).

## The enigmatic and non-canonical roles of caspases

During evolution, nature selects genes and their products that, in different situations and intracellular compartments, can perform diverse functions. Indeed, recent research has shed light on the multifunctional roles of caspases, which had traditionally been recognized for their crucial involvement in PCD. However, emerging evidence indicates that these proteolytic enzymes are utilized for a plethora of non-apoptotic roles, which are often crucial for vital processes [34]. Investigations into the function of individual caspases, conducted using caspase knockout mice, have revealed that these enzymes contribute substantially to the regulation of various cellular activities, including cell proliferation, differentiation, migration, invasion, and inflammation [35, 36].

### Caspases as drivers of proliferation

**Fig. 2:**
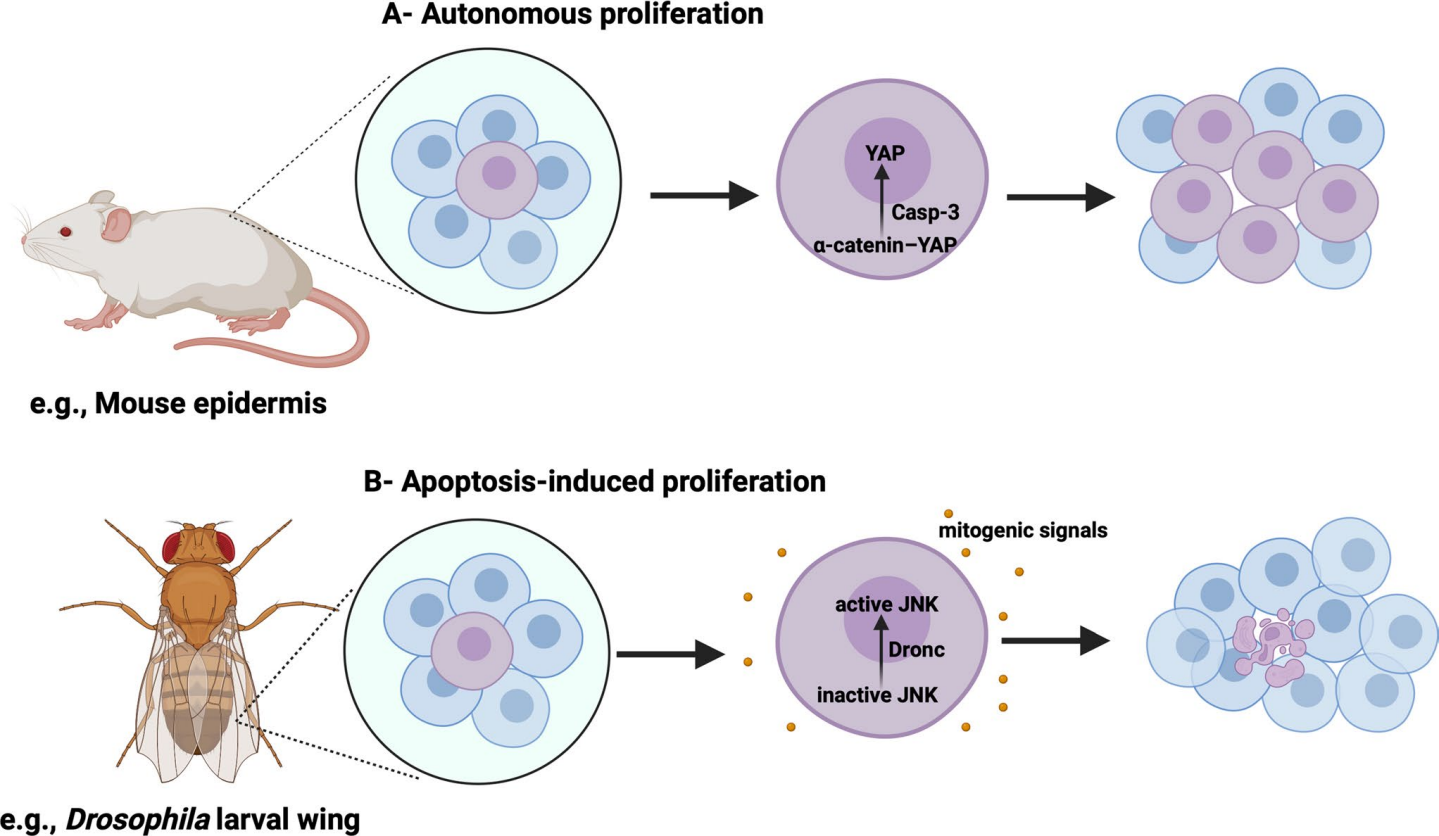
Caspases induced proliferation

The involvement of caspases in triggering proliferation is remarkable: Paradoxically, enzymes capable of cell destruction can also stimulate cell division. How do caspases drive cell proliferation? There are different scenarios where caspase-induced proliferation has been proven [2]. According to the first scenario, autonomous proliferation appears when a cell in which caspases have been activated exhibits enhanced proliferation. Sublethal activation of caspase-3 regulates cell proliferation by the proteolytic cleavage of a variety of substrates including regulators of cell cycle checkpoints, such as retinoblastoma (Rb) and p21 [37]. Additional studies revealed that the cleavage of these substrates by caspases regulates the cell cycle’s progression, even without PCD. In the mouse epidermis, caspase-3 knockout results in impaired growth of epidermal cells and a decrease in the size of the sebaceous gland. This indicates that the maintenance of these cells relies on caspase-3. In addition, caspase-3 cleaves α-catenin in mouse epidermal cells, leading to the release of yes-associated protein (YAP) from the α-catenin–YAP complex. This activation enables YAP to translocate into the nucleus and drive a proliferative response [38] (Fig. 2A). Once YAP translocates to the nucleus, it drives the expression of a variety of target genes involved in the control of S-phase entry and mitosis. Thus, YAP might fulfill the function of a regulator of cell proliferation and organ size. To confirm these results, researchers examined the activation of YAP-dependent target genes in HaCaT cells in the presence of z-DEVD-fmk, a specific caspase-3 inhibitor. HOXC13, a key player in the formation of hair follicles, showed a 5-fold decrease in gene expression in z-DEVD-fmk-treated cells. Similarly, HOXA5, known for its ability to suppress stem cell characteristics by blocking Wnt signaling, exhibited a 2-fold decrease in gene expression in the same cells. In addition, the expression of the receptor tyrosine kinase ErbB4 was reduced by 6-fold, and the expression of WTIP, a member of the Ajuba family and an inhibitor of the Hippo pathway, was reduced by 2-fold. These results demonstrate that the activity of YAP and a linked set of processes are significantly reduced when caspase-3 is inhibited [38].

Caspase-3 and/or caspase-7 play a critical role in cell proliferation in various cancer cell lines, including HeLa, HepG2, and Jurkat. To further investigate the caspase effect on proliferation, researchers tested subtype-specific caspase inhibitors against caspase-3, caspase-7, caspase-8, and caspase-9 separately in HepG2 cells. Caspase-8 inhibition slightly prevented the proliferation of these cells, while caspase-3 and/or caspase-7 inhibition effectively prevented proliferation. On the other hand, caspase-9 inhibition did not affect proliferation. The authors also investigated the effect of caspases on cell cycle regulation. Caspase-7 knockout, but not caspase-3 knockout, in HeLa cells caused cell cycle arrest at the mitotic phase, suggesting that the regulation of the cell cycle progression at the mitotic phase is mainly due to caspase-7 activity. Because caspase-8 is the upstream-activated enzyme in the processing and activation of caspase-3 and caspase-7 in apoptotic cells, it may also contribute to cell cycle regulation through caspase-7 [39]. On the contrary, mouse embryonic fibroblasts (MEFs) with caspase-2 knockout showed a higher proliferation rate, and the mouse Eµ-Myc lymphoma model with loss of caspase-2 showed accelerated tumorigenesis. In addition, caspase-2 deficiency has been associated with impaired cell cycle arrest in response to DNA damage [40, 41]. This implies an important function of caspase-2 in cell cycle arrest or cell cycle checkpoint regulation.

Caspases also have a role in T cell proliferation. The levels of caspase-3 and caspase-8 are associated with the timing of activated mouse T cells entering the proliferative phase, as indicated by the expression Ki67. In addition, the use of caspase inhibitors suppresses T cell proliferation [42, 43]. Caspases also contribute to regulating the proliferation of mouse myocytes. The hearts of neonatal mice with a cardiac-specific double mutation of caspase-3 and caspase-7 show cardiomyocyte hypertrophy and fewer myocytes compared with wild-type (wt) mice. To gain insight into how caspase-3 and caspase-7 knockout reduce the number of cardiomyocytes, the authors performed microarray-based gene expression analysis. Newborn caspase-deficient myocardium transcribes abnormally low levels of genes encoding key proteins involved in DNA replication, recombination and repair, centromere and kinetochore formation, and cell cycle regulation. In young hearts, lack of caspase-3 and caspase-7 during development results in abnormally high expression of genes involved in cell cycle inhibition, genes coding for non-cardiac isoforms of sarcomeres’ proteins, and slightly reduced expression of genes encoding components of the contraction machinery. To confirm that this proliferative effect is independent of the caspase proteolytic activity, the authors used postnatal 4 (P4)–P5 rat postnatal myocytes that overexpressed wt caspase-3 and caspase-7 or caspase mutants bearing a cysteine to serine substitution in the catalytic site (mutant caspase with a lack caspase proteolytic activity). Overexpression of normal zymogens or mutants results in comparable upregulation of genes that are suppressed in the caspase-deficient myocytes, providing evidence that caspases directly influence the regulation of genes involved in regulating myocyte proliferation [44].

The second scenario of caspase-driven proliferation is linked to apoptosis-induced proliferation (AiP). Dying cells can emit mitogenic signals that promote proliferation in a non-autonomous manner [2]. In this situation, caspases are essential in both the destruction of the cell in which they are activated and the proliferation of the neighboring cells. AiP is crucial for homeostasis, promoting wound healing, and regenerating tissues in different organisms, spanning from flatworms to mammals [45]. In the 1970s, researchers first documented that *Drosophila* larval wing epithelial tissue has the ability to regenerate after induction of cell death by X-ray radiation. This regeneration occurs through cell proliferation, ultimately leading to the development of adult flies with normal wings [46]. Under pathological conditions when the execution of apoptosis is compromised and the stressed cells are kept alive, continuously active AiP promotes tissue overgrowth. Although the mechanisms by which caspases activate AiP are not well understood, it appears that c-Jun N-terminal kinase (JNK) is involved in a downstream effector step. In the proliferating tissues of the *Drosophila*, the initiator caspase Dronc activates JNK, which leads to the release of mitogens including Wingless, Decapentaplegic, *Drosophila* Wnt, and others (Fig. 2B). Another mechanism could be that Dronc associates with Duox, an NADPH oxidase, at the plasma membrane and stimulates the production of reactive oxygen species (ROS), which promote AiP [46]. Thus, certain caspases, such as caspase-3, caspase-7, and caspase-8, have a vital role in promoting cell proliferation, which can affect the same cell that activates caspases or the neighboring cells. However, the detailed mechanism has yet to be revealed, and the role of caspases in cell cycle regulation is still controversial in terms of whether caspases are cell cycle accelerators or brakes.

There are numerous examples regarding the impact of caspase activity on cell cycle transition, specifically in the hematopoietic system. In B cells, co-stimulation via cluster of differentiation 40 (CD40) and CD180 results in the cleavage and activation of caspase-6 and caspase-8 in a time-dependent manner, reaching a peak at 12 and 24 h after stimulation, respectively. The rate of proliferation induced by either CD40/CD180 or lipopolysaccharide (LPS) stimulation could be decreased significantly by chemically inhibiting either caspase [47]. Cleavage of the transcriptional repressor SATB1 by caspase-6 activates transcription of cyclin D1/2 and CDK4, which in turn promotes the exit from the G_0_ phase. It is important to note that the failure of cell growth could be partially due to non-cell-cycle effects, such as activation of necroptosis after prolonged suppression of caspase-8.

On the other hand, previous research revealed that caspase-3 is a suppressor of B cell proliferation. Mice lacking caspase-3 exhibit an enlarged spleen and lymph nodes, characterized by elevated B cell counts and enhanced proliferation. Interestingly, the excessive cell growth observed in this case is controlled by the cell-cycle inhibitor CDKN1A/p21. Mice lacking the caspase-3 gene have elevated levels of p21, and when both caspase-3 and p21 are deleted, the excessive cell growth phenotype is eliminated [3].

**Fig. 3:**
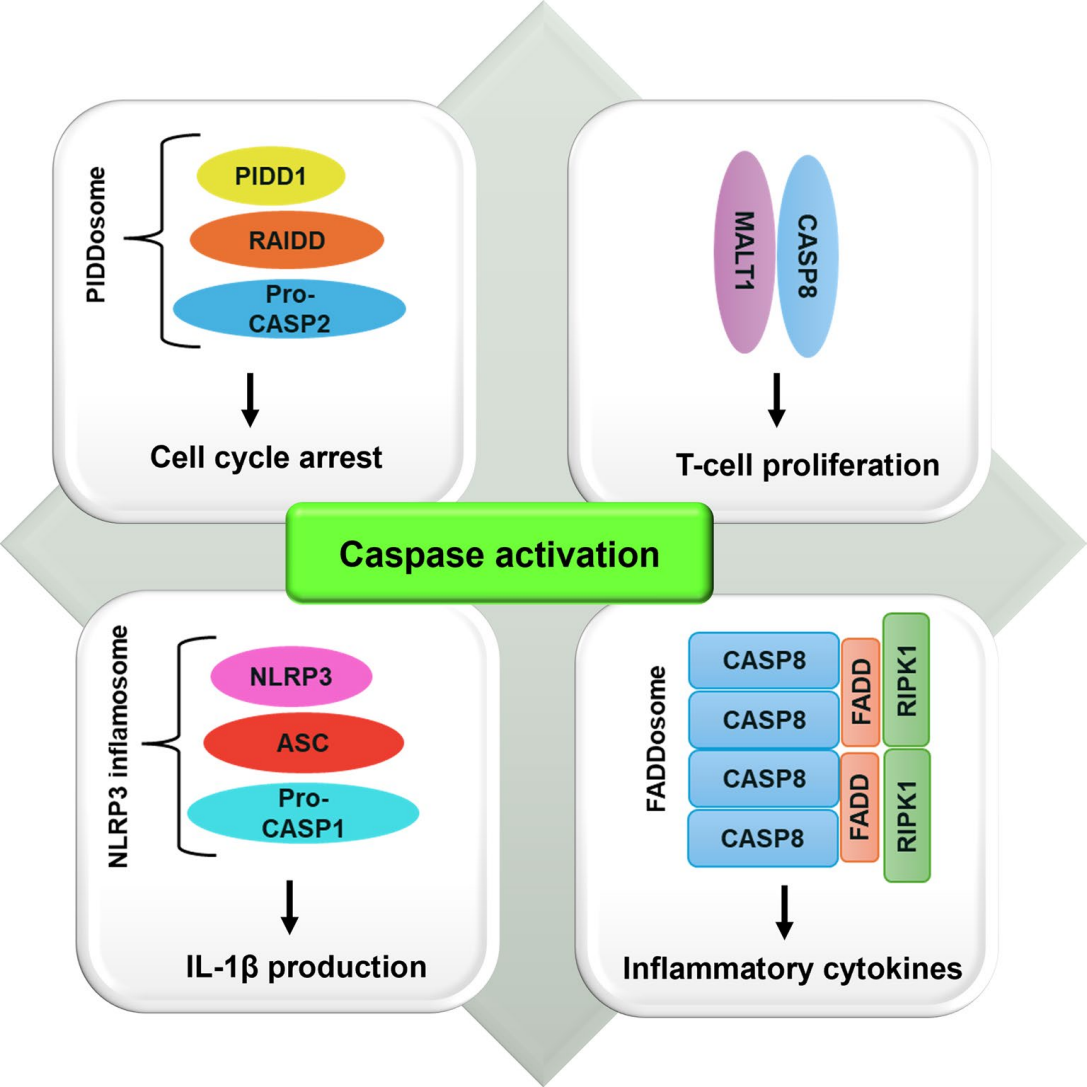
Caspase activation via complex assembly

Caspase-2 also has a role in the cell cycle. The caspase-2–PIDDosome complex (Fig. 3) promotes G_1_-phase cell cycle arrest. An active caspase-2–PIDDosome complex leads to specific cleavage of the E3 ubiquitin ligase Mdm2 at Asp 367. This results in the loss of the C-terminal RING domain, which is critical for the ubiquitination of p53. The N-terminally truncated form of Mdm2 interacts with p53 and enhances its stability, which can result in cell cycle arrest and senescence by upregulating p21 expression [48].

Caspase-8 has been suggested as a regulator of S-phase entry in vitro and in vivo. Indeed, caspase-8 knockout T cells show defective S-phase entry upon CD3/CD28 co-stimulation. These T cells remain in the G_0_/G_1_ phase of the cell cycle upon CD3 stimulation and fail to undergo CDK1/2 or cyclin A upregulation or phosphorylation of Rb. Caspase-8 may also participate in T cell proliferation via the formation of a heterodimer of caspase-8 and the paracaspase MALT1 (Fig. 3), which facilitates BCL10-driven nuclear factor kappa-light-chain-enhancer of activated B cells (NF-κB) activation and IL-2 transcription essential for T cell proliferation [3].

### Caspases in cell differentiation

Cellular differentiation is a process during which a cell changes its gene expression to become a more specialized cell type. This remarkable transformation allows multicellular organisms to develop cells with distinct functions. A common characteristic between apoptosis and differentiation is nuclear disruption, which is an essential stage leading to cell death. Similarly, the terminal differentiation of certain cell types, including lens fiber cells and erythrocytes, is marked by complete nuclear deletion [49]. This unique change in cellular morphology suggests that caspase activation may regulate both PCD and differentiation. Consistently, blocking caspase-3 and caspase-1 activation can inhibit nuclear degradation by reducing poly (ADP)ribose polymerase (PARP) cleavage in lens fiber cells [50, 51]. During erythrocyte maturation, cells require transient activation of various caspases. For example, caspase-3 activation enhances PARP cleavage [52]. Additionally, caspase-6, an effector caspase, which might be activated by caspase-3, cleaves proteins essential for maintaining nuclear integrity (such as lamin B) and facilitating chromatin condensation (such as acinus) [53].

Besides changes in nuclear morphology, other common adaptations associated with apoptosis have been observed consistently in differentiating cells. Caspase-3 activation has been reported to induce apoptosis-like features during the differentiation of megakaryocytes, myoblasts, osteoblasts, and neurons. For example, caspase-3 can trigger platelet microparticle formation, which is morphologically similar to the membrane blebbing process of apoptosis [54]. In skeletal muscle, actin filament rearrangement occurs in both cell death and differentiation [55]. Furthermore, myosin light chain kinase, a muscle protein essential for muscle contraction, is also required for typical apoptotic membrane blebbing [56]. An increase in matrix metalloproteinase activity is key to facilitating the membrane fusion observed in both muscle cell differentiation and apoptosis [57, 58]. These events suggest that myoblast differentiation and apoptosis might utilize similar pathways. Caspase-3 activation can trigger myoblast fusion and myotube formation by targeting the mammalian sterile twenty-like kinase (MST1) [59]. Besides, the bone remodeling process requires balancing osteogenic cell death and differentiation of osteoblasts, processes that are regulated by caspase activity [60, 61]. Caspase-3 activation mediates the differentiation of bone marrow (BM) stromal stem cells, with an upregulation of the transforming growth factor beta (TGF-β)/Smad2 signaling pathway [62]. Moreover, neuronal differentiation typically involves neurite extension, which relies on substantial cytoskeletal changes like those observed during apoptosis. Caspase-3 may mediate the activation of the transcription factor myocyte enhancer factor 2 (MEF2), a protein associated with the neuronal survival pathway [63, 64]. According to another study, caspase-3-mediated rho-associated, coiled-coil-containing protein kinase 1 (ROCK1) can trigger either apoptosis or neuronal differentiation [65]. In embryonic keratinocytes, caspase-3 cleaves protein kinase C-delta (PKC-δ) and is involved in terminal differentiation. Notably, this event was observed more frequently in embryonic keratinocytes than in keratinocytes derived from newborns [66], suggesting that timing is crucial for cell fate.

In addition to the apoptotic and inflammatory caspases involved in the process of differentiation, caspase-14 plays a unique role in cellular differentiation, especially in the skin. Caspase-14 is predominantly expressed in the epidermis and is crucial for the formation of the skin barrier [67, 68]. During keratinocyte differentiation, caspase-14 cleaves profilaggrin into filaggrin. This process is pivotal in the formation of the cornified envelope, which provides the skin with structural integrity, hydration, and protection against ultraviolet (UV) radiation [69]. Experimental evidence indicates that mice deficient in caspase-14 are highly susceptible to UVB-induced epidermal dehydration [70]. This suggests a loss of the stratum corneum’s UVB-filtering capacity due to a deficit in keratinocyte differentiation.

Overall, the activation of caspases is a critical event in cellular differentiation. Their involvement in differentiation reveals the versatility and complexity of these enzymes. The precise timing and cell-specific activation of caspases are vital for organisms to develop correctly, highlighting the deep connection between cell death and differentiation pathways.

### Caspases in cell migration and invasion

Cell migration is essential for the regulation of numerous biological processes, including embryonic development, the immune response, and wound healing. However, it is also a critical contributor to pathological conditions such as tumor invasion and metastasis. Accumulating evidence shows that caspases have been linked to cell migration and motility. The role of caspase-8 in cell migration has been investigated in mice lacking the caspase-8 gene. These knockout mice fail to assemble a functional circulatory system, suggesting a defect in endothelial cell migration that results in early embryonic lethality [71–73]. Several studies also suggest that the expression of caspase-8 in endothelial cells is required for the formation of a proper vascular system. Consistently, endothelial cell-specific caspase-8 knockout mice show reduced postnatal retinal angiogenesis [74]. The genetic depletion or inhibition of caspase-8 decreases the expression of the integrin subunit α5 and the chemokine receptor CXCR4 in endothelial progenitor cells, resulting in inhibition of cell adhesion and migration, finally reducing neovascularization [75]. Moreover, the non-apoptotic functions of caspase-3 have been determined using caspase-3 knockout mice compared with wt mice. At 8–12 months of age, the surviving caspase-3 knockout mice show glomerular lesions [76]. Investigation of the involvement of caspase-3 in kidney development demonstrated that caspase-3 regulates ureteric branching by promoting cell migration of ureteric bud cells [77]. Caspase-9 directly cleaves semaphorin 7 A (Sema7A), subsequently promoting axonal guidance and neuronal migration in neural development [78]. In caspase-9 and apoptotic peptidase activating factor 1 (Apaf-1) mutant mice show misrouted axons, impaired synaptic formation, and defects in olfactory sensory neuron maturation [79]. Recently, researchers reported a novel function of caspase-1 in facilitating the migration of hair follicle stem cells into the epidermis through the activation of CARD [80]. Collectively, the non-apoptotic functions of caspases, particularly caspase-8, caspase-3, caspase-9, and caspase-1, are crucial for various cell migration processes. Experimental evidence suggests that deficiency of any of these caspases leads to abnormal development of the circulatory, renal, neuronal, and integumentary systems, shedding light on the significance of these caspases in proper cellular movement and organogenesis.

In addition to the migratory roles of caspases in cellular development and the immune response, caspases also promote tumor development via their ability to modulate migration and invasion. For example, activation of caspase-8 by phosphorylation at Tyr-380 can induce an interaction between caspase-8 and the p85α subunit of phosphoinositide 3-kinase (PI3K), resulting in increased cell adhesion and motility [81]. Besides, activation of caspase-3 has been reported to promote migration and invasion of at least colorectal, skin, and ovarian cancers. A study of the molecular mechanism of caspase-3 in the promotion of ovarian cancer cell migration showed that this enzyme is activated by the stimulation of laminin-10/11 through β1 integrin signaling. This activation leads to cleavage and activation of calcium (Ca^2+^)-independent phospholipase A2 (iPLA2), resulting in the production of lysophosphatidic acid (LPA) [82]. LPA, in turn, activates its receptor and triggers downstream signaling pathways, including PI3K/extracellular signal-regulated kinase (ERK)/p38 mitogen-activated protein kinase (MAPK)/Akt and PI3K/cytosolic PLA2 (cPLA2) pathways [83, 84]. cPLA2 is crucial for LPA-induced production of arachidonic acid, which is a primary mediator of cell migration [84]. Caspases can also regulate the migration of brain tumors. Indeed, caspase-3 and caspase-8 can cleave gelsolin, a protein heavily involved in cell motility, and promote migration and invasiveness in glioblastoma cells [85]. The accumulated data indicates that the activation of caspases triggers a cascade of molecular signaling pathways that enhance cell migration and invasion. Understanding these mechanisms not only highlights the pro-tumorigenic roles of caspases but also opens new avenues for studying cancer metastasis.

### Caspases in inflammation/infection

As mentioned above, inflammatory caspases influence the innate immune response as well as IL-1β and IL-18 production. Activating the latent pore-forming protein gasdermin D (GSDMD) causes plasma membrane rupture and cell lysis via pyroptosis, a pro-inflammatory cell death mechanism [86]. This fact raises the question of whether inflammatory caspases have a role beyond pyroptosis. These enzymes regulate antimicrobial cell activities. Caspase-1 controls phagosome maturation during gram-negative and gram-positive bacterial infections. It also promotes fusion between vacuoles containing *Legionella pneumophila* and lysosomes. In BM-derived macrophages infected with *L. pneumophila*, caspase-1 is activated by the nucleotide-binding domain, leucine-rich containing (NLR) protein Ipaf (Fig. 3). Upon activation, caspase-1 cleaves pro-IL-1β, pro-IL-18, and IL-33 to generate biologically active cytokines. However, the addition of IL-1β, and/or IL-18 to macrophages at the time of infection did not suppress *Legionella* replication in Ipaf knockout macrophages, indicating that caspase-1 activation is critical for the clearance of the bacterium independently of the activity of IL-1β and IL-18. It is more likely that caspase-1 plays a role in regulating the maturation of the *Legionella*-containing phagosome (LCP), as the fusion of the LCP with lysosomes is essential to restrict the replication of *Legionella* in wt macrophages [87]. The exact mechanism of how caspase-1 controls phagosome formation is not fully understood; however, it is possible that caspase-1 acts by targeting and processing host proteins involved in the formation and/or transport of the LCP.

In macrophages, caspase-1 modulates the pH of phagosomes in gram-positive bacteria such as *Staphylococcus aureus*, enhancing pathogen death. In phagocytosis-associated caspase-1 activation, the most specific caspase-1 inhibitor, YVAD, suppresses acidification of *S. aureus*–containing vacuoles. Caspase-1 can modulate the pH of phagosomes by hydrolyzing the components of the NOX2 complex, which results in restriction of NOX2 activity, ROS production in the phagosome, and, hence, control of vacuolar pH [88]. Moreover, caspase-1 and caspase-11 activities are both crucial for inhibiting the intracellular growth of *Salmonella Typhimurium* independently of or before the onset of cell death. The lack of GSDMD does not effectively reduce growth attenuation for up to 10 h. In addition, the lack of the adaptor protein apoptosis-associated speck-like protein containing a CARD (ASC), the cytokine IL-18, or the IL-1β receptor (IL-1R) did not result in enhanced proliferation of cytosolic bacteria, suggesting a role other than pyroptosis [89]. The precise mechanism has not yet been fully elucidated; however, as described earlier, caspase-1 might regulate the acidification of macrophage phagosomes. Additionally, caspase-11 has additional roles, such as promoting the fusion of *Legionella* vacuoles with lysosomes by modulating cofilin, a protein that regulates actin polymerization. Thus, it might have similar activities in *S. Typhimurium* [90]. It is known that sublethal activation of caspases results in mitochondrial proinflammatory activity. During bacterial/viral infection, caspase-activated DNase (CAD) is activated in a caspase-dependent manner. CAD activation led to the secretion of IL-8 and CXCL1, IL-6, and IFN type III (IFN-λ) via activation of NF-κB and cGAS/STING pathways. Infection of CAD-deficient HeLa cells with DNA virus such as Herpes simplex virus 1, or an RNA virus such as influenza A virus, results in low secretion of IL-6 levels and interferon-responsive genes MX1and IFI44L compared to wt HeLa cells [91]. Caspase-11 influences immune cell migration, allowing cells to navigate to sites of infection or injury. Caspase-11-deficient lymphocytes exhibit a cell-autonomous migration defect, suggesting that caspase-11 is directly involved in regulating cell movement. The CARD of caspase-11 interacts with the carboxy-terminal WD40 propeller domain of actin-interacting protein 1 (Aip1), promoting cofilin-mediated actin depolymerization [92]. Notably, caspase-11 activity is tightly regulated to prevent dysregulation of enzymes, which can cause excessive inflammation and tissue damage. During infections, caspase-11 facilitates cell migration, aiding the rapid response to pathogens. Conversely, in chronic inflammatory diseases, caspase-11 may exacerbate pathology by promoting persistent inflammation. Previous studies using caspase-11 knockout mice demonstrated reduced neutrophil migration into joints and atherosclerotic lesions, offering protection against inflammatory diseases such as arthritis [93] and atherosclerosis [94].

A further area of inquiry is whether caspases function in the inflammatory tumor microenvironment (TME). The involvement of caspase-8 in the expression of cytokines and chemokines has been observed in several cancer cells. TNF-related apoptosis-inducing ligand (TRAIL) receptors (TRAIL-Rs) are typically linked to caspase-8-mediated cell death. However, TRAIL-Rs can stimulate the generation of pro-inflammatory cytokines through caspase-8. The production of cytokines through the TRAIL pathway necessitates the presence of caspase-8 in the FADDosome (Fig. 3), although its activity is not required. The FADDosome is composed of FAS-associated death domain protein (FADD), caspase-8, and RIPK1. When caspase-8 proteolytic activity is inhibited, apoptosis is prevented, but the release of cytokines mediated by the FADDosome is not affected in the presence of TRAIL. However, if caspase-8 is knocked down or completely deleted, both apoptosis and cytokine release are blocked. Catalytically inactive caspase-8 mutants (e.g., D216A, G325A, and D210A) cannot restore the apoptotic pathway, but they effectively mediate cytokine expression by increasing NF-κB activation. This phenomenon explains why certain types of cancer promote the expression of mutated caspase-8 and how they even derive advantages from the expression of TRAIL-Rs [95]. Caspase-8 is also involved in the regulation of inflammatory processes and insulin sensitivity in adipocytes [96]. This is more likely due to the utility of caspase-8 in the production of cytokines through the FADDosome and its participation in the regulation of adipocyte metabolism, through the cleavage and inactivation of peroxisome proliferator-activated receptor gamma resulting in decreased lipogenesis and glucose uptake [96]. In addition, as mentioned above, caspase-8 can directly cleave pro-IL-1β or interact with the NLR3 inflammasome, promoting the indirect activation of IL-1β. In BM-derived cells, caspase-8 was able to increase the level of inflammatory factors such as IL-1β, IL-6, and C-X-C motif chemokine ligand 10 (CXCL10) through Toll-like receptor 3 (TLR3) and TLR4. Inhibition of caspase-8 led to higher levels of anti-inflammatory cytokines and chemokines, suggesting that caspase-8 is crucial for the regulation of inflammation. Nevertheless, it is important to investigate whether caspase-8 regulates the inflammatory TME to promote or suppress tumor growth [95]. Furthermore, in the case of ovarian cancer, there is a direct correlation between low caspase-8 expression and the presence of chronic inflammation and immune resistance. This correlation ultimately contributes to tumor aggressiveness. Indeed, caspase-8 has an antitumorigenic effect on the primary tumor cells and the TME by regulating the activation of B and T lymphocytes, as well as the differentiation and polarization of macrophages [97]. Caspase-1 also contributes to the TME progression. In triple-negative and basal-like breast cancer cells caspase-1 is activated within the inflammasome, a complex formed by the assembly of caspase-1, Nod-like proteins (NALP), and ASC (Fig. 3). Activated caspase-1 promotes proteolytic cleavage of pro-IL-1β, allowing IL-1β maturation, and tumor-associated macrophages recruitment to the TME and, therefore, tumor progression [98, 99]. Thus, caspase-mediated non-apoptotic functions are crucial in TME.

## The nonconformist story of caspase deregulation

**Fig. 4:**
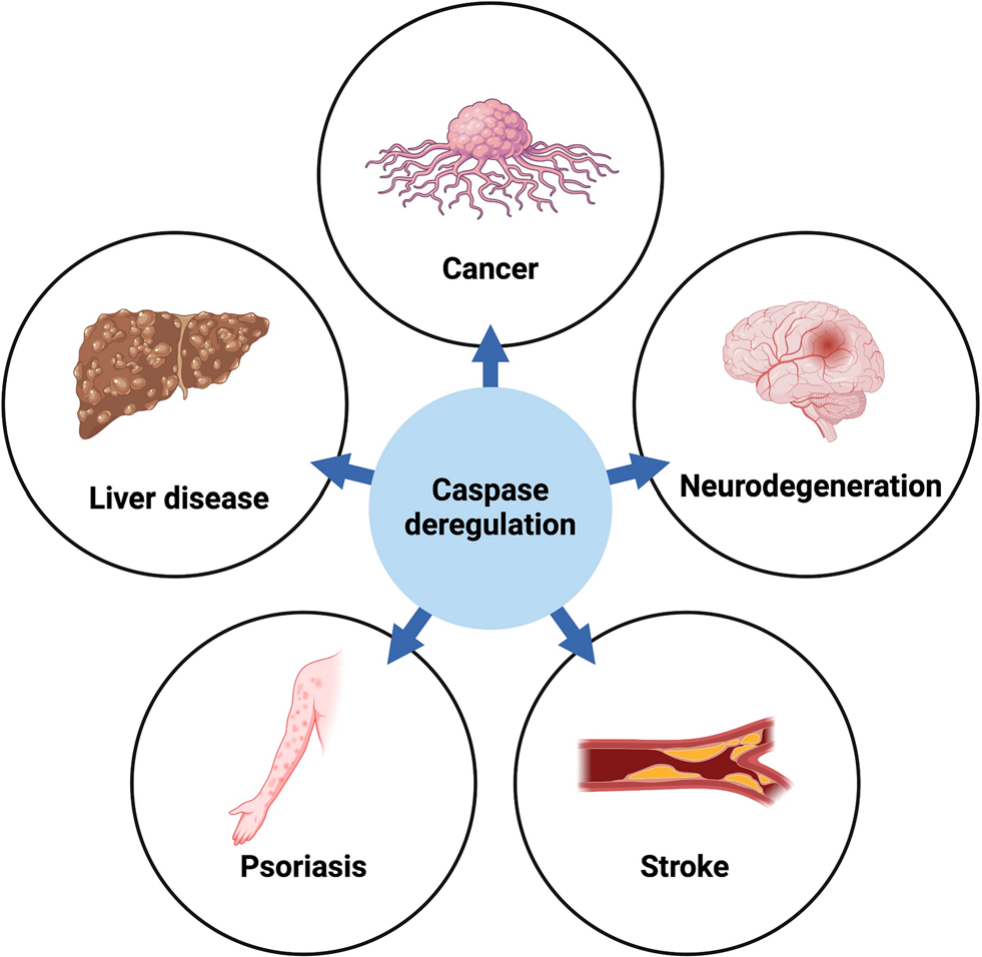
Caspase deregulation and disease

Caspase-dependent apoptosis has received a lot of attention due to its implication in a myriad of diseases. However, it is important to differentiate the effects exclusively related to the apoptotic activity of caspases and the outcomes resulting from their non-apoptotic functions (Fig. 4). Below, we discuss the intriguing link between the non-apoptotic functions of caspases and disease.

### Cancers

Based on the current knowledge, apoptotic caspases are linked to a defensive mechanism against tumorigenesis. On the other hand, several studies have addressed the non-lethal functions of caspases that can support tumor formation and development. Based on the non-cell death utilities of caspases described above, it is evident that the activation of caspases can induce cell proliferation, migration, and inflammation. These non-cell death activities assist cancer cells in rapid growth and becoming more aggressive.

Caspase-3 overexpression has been observed in various cancers, including hepatocellular [100] and lung [101] carcinomas. The increased caspase-3 expression in these tumor cells underscores the importance of its non-cell death functions in tumor cell formation. The caspase-3 functions in tumorigenesis can be categorized into two mechanisms: nonautonomous and cell-autonomous. For the nonautonomous mechanism, apoptotic cells induce adjacent cells to proliferate, a phenomenon described above as compensatory proliferation or AiP. This process commonly occurs in tissue regeneration [102, 103]. However, the nonautonomous roles of caspase-3 can also lead to abnormal proliferation of malignant cells, contributing to tumor repopulation, therapeutic resistance, and angiogenesis [104–106]. In the cell-autonomous mechanism, caspase-3 is intrinsically regulated within the tumor cells. This regulation occurs through the proteolytic cleavage of various substrates, ultimately influencing cell cycle progression, cell differentiation, and tumorigenesis [43].

High caspase-7 expression is associated with a poor prognosis in patients with oral squamous cell carcinomas [107]. Furthermore, caspase-7 overexpression has been observed in breast carcinoma tissues and is correlated with increased levels of estrogen receptor-alpha (ERα), indicating enhanced transcriptional regulation of caspase-7 by the ERα transcription factor in breast cancer cells. Additionally, activation of caspase-7 promotes breast cancer cell growth and proliferation by downregulating the p21^Cip^ protein, a potent inhibitor of cyclin-dependent kinases (Cdks) [108]. Caspase-7-deficient Chinese hamster ovary cancer cells as well as HeLa ovarian cancer cells are characterized by a reduced cell proliferation rate due to cell cycle arrest at the G_2_/M phase [39, 109]. These findings suggest that the non-apoptotic roles of caspase-7, particularly in cell proliferation, contribute to tumor cell growth and ultimately lead to increased tumor aggressiveness in patients.

Caspase-8 expression in tumor cells varies across different cancer types. Researchers have reported discrepancies in caspase-8 levels in specific tumors [97, 110, 111]. Moreover, analyses that predict the relationship between caspase-8 expression and patient survival outcomes suggest that high levels of caspase-8 may correlate with either a poor or favorable prognosis, depending on the type of cancer [97]. Hence, caspase-8 can act as both a promoter and inhibitor of tumor growth. Caspase-8 upregulation in cancerous cells is often associated with abnormal activation of molecular mechanisms. These mechanisms inhibit the complete activation of caspase-8’s proteolytic function, which is responsible for triggering apoptosis [97]. Besides, activation of caspase-8 can mediate non-apoptotic roles such as cell adhesion, migration, inflammation, and cell cycle control, which influence cancer cell progression (as described above).

In non-small cell lung cancer (NSCLC), there is a high expression of caspase-9b, an alternatively spliced short isoform of caspase-9 [112]. This protein lacks catalytic function and exhibits oncogenic activity by inhibiting the formation of the apoptosome complex with caspase-9 [113, 114]. Furthermore, the non-apoptotic functions of caspase-9 have been explored in acute myeloid leukemia, demonstrating that activation of caspase-9 activity promotes granulocytic differentiation in leukemic cells [115].

Caspase-2 is widely recognized as a tumor-suppressive protein due to its role in apoptosis. Beyond its apoptotic activity, caspase-2 regulates non-apoptotic functions, such as the cell cycle checkpoint. In polyploid cells, the cleaved fragment of PIDD1, PIDD1-CC, recruits RAIDD and pro-caspase-2 to form the PIDDosome complex, resulting in caspase-2-mediated Mdm2 cleavage, p53 stabilization, and p21-mediated cell cycle arrest [33, 116]. This scenario demonstrates the onco-protective effect through the activation of caspase-2. Conversely, recent research has revealed that caspase-2 may inhibit ferroptosis in p53-mutant lung and esophageal cancer cells. This inhibition is achieved by suppressing the chaperone-mediated autophagic degradation of glutathione peroxidase 4 (GPX4), an enzyme essential for preventing ferroptosis. Notably, the inhibitory effect of caspase-2 on ferroptosis is independent of its canonical proteolytic activity [22]. These findings suggest that caspase-2 could exhibit both tumor-suppressive and oncogenic activities, depending on the genetic background of the tumor cells. Thus, the dysregulation of non-cell death-associated functions of caspases can exacerbate tumor formation and disease progression.

### Neurodegenerative diseases

A variety of neurodegenerative diseases including Alzheimer’s disease (AD), Huntington’s disease (HD), and Parkinson’s disease (PD) have been associated with deregulated activation of caspases, along with other changes. Accumulating evidence suggests that caspases have a non-apoptotic function in the brain. Consistently, caspase-1 activation has been shown to be associated with age-dependent and cognitive impairment in humans. Caspase-1-null J20 mice are protected from episodic and spatial memory deficits, neuroinflammation, and amyloid-β (Aβ) accumulation [117]. Several other studies have revealed that the aggregation of specific proteins, such as Aβ peptide, the microtubule-stabilizing protein (tau), or mutant huntingtin (mHtt), are associated with elevated levels of caspase-2 in the brain and its neuronal activity. Active caspase-2 cleaves aggregate-forming proteins and regulates dendritic spine density, promoting the pathogenesis of neurodegenerative diseases [118]. Interestingly, caspase-2 and Δtau314 are upregulated in the brain tissues of patients with AD or cognitive impairment compared with healthy individuals. Decreasing the level of caspase-2 in mice diminishes the formation of Δtau314 and reverses existing memory deficits. As in AD, there are elevated levels of caspase-2 and Δtau314 in the prefrontal cortex and striatum of patients with HD compared with healthy individuals. Both Δtau314 and caspase-2 levels correlate positively in these brain structures. On the other hand, progressive synaptic degeneration and neuron loss are major structural correlates of cognitive impairment in AD. Patients with AD exhibit elevated procaspase-3 and active caspase-3 levels that are most evident in the postsynaptic density fractions [119]. Although the mechanism of the procaspase-3 increase is not clear, it is very likely that the endogenous level of procaspase-3 increases at the beginning and early stages before caspase-3 activation [120]. Indeed, expression of procaspase-8 and procaspase-3 in AD brains and the caspase-8/RIPK3 axis are critical for promoting both Aβ deposition and gliosis in the 5xFAD mouse model of AD. Caspase-8 is responsible for the regulation of Aβ-driven inflammasome gene expression and IL-1β release. On the other hand, amyloid precursor protein (APP), the parent protein of Aβ, is cleaved by caspase-3, which correlates with synaptic loss and cognitive dysfunction. Furthermore, caspase-3 inhibition decreases APP cleavage [121]. Activated caspase-3 is observed in hippocampal dendritic spines of an AD murine model concomitant with the onset of memory decline and without obvious cell death. Activation of caspase-3 is also associated with the pathogenesis of PD [122].

Active caspase-6 is present in postmortem brains of patients with HD and AD that do not display apoptotic morphology. Caspase-6 has an important role in axonal degeneration, which makes a profound contribution to neuronal loss in HD and AD. Moreover, caspase-6 is considered to be an upstream modulator of AD pathogenesis as active caspase-6 is abundant in neuropil threads, neuritic plaques, and neurofibrillary tangles in AD brains [123]. Moreover, caspase-6 can cleave tau, and the truncated form of this protein may interfere with the assembly of neuronal microtubules and inhibit the transportation of structural components, vesicles, and organelles to distant areas of the neuron [124]. Therefore, in AD, neurons may undergo dysfunction even in the absence of cellular demise. This implies clear evidence that the deregulation of caspases is involved in neurodegenerative pathologies. There are still unresolved questions about how the caspases are deregulated in the above-discussed diseases.

There is evidence that the mitochondrial apoptotic pathway is central to many of the non-apoptotic processes in the brain. Consistently, in long-term depression (LTD), active caspase-3 is present in the postsynaptic structure and is associated with the release of cytochrome *c*. These data suggest that the mitochondrial pathway plays a non-apoptotic role in LTD [125]. There are also less well-understood mechanisms involving changes in intracellular Ca^2+^ concentrations. The changes in Ca^2+^ levels that occur during neuronal cell differentiation are linked to caspase activation. The precise mechanism by which caspases are activated remains unknown. The involvement of the low-voltage-dependent T-type Ca^2+^ channel that increases the Ca^2+^ levels that are required to activate caspase-3-dependent neurogenesis during the development of the cortex has been shown [125]. These pathways seem likely to be linked to several different neurodegenerative diseases such as long-term depression, Amyotrophic Lateral Sclerosis (ALS), AD, and other conditions.

### Other disorders

The deregulation of caspases is not only confined to cancer and neurodegeneration. Several reports indicate that there is an association between caspase activation and liver disease. In the liver lobules of patients infected by hepatitis C virus (HCV), caspase activation is considerably elevated compared with normal controls; importantly, the immunoreactive cells do not show apoptotic morphology [126]. The extent of caspase activation correlates significantly with the disease grade. In biopsy specimens with low activity (grade 0), 7.7% of the hepatocytes revealed caspase-3 activation, and the staining of 20.9% of the cells indicated grade 3. A mouse model of non-alcoholic steatohepatitis (NASH) revealed marked caspase-3 activation in hepatocytes, in conjunction with steatohepatitis and increased hepatic triglyceride levels, liver inflammation, and fibrosis. Caspase-3 activity is mediated by CCR2-dependent infiltration of Ly6c-positive monocytes rather than apoptosis [127]. Likewise, caspase-2 protein expression is strongly localized to injured hepatocytes, correlating with NASH severity. Caspase-2 has been linked to lipoapoptosis, but is apoptosis the sole determinant of increasing the severity of NASH, or can it be anti-inflammatory and linked to other metabolic activity of caspase-2? Caspase-2-deficient mice fed a high-fat diet are protected from abdominal fat deposition, dyslipidemia, and hepatic steatosis. Adipose tissue in caspase-2-deficient mice is more proliferative, with upregulation to mitochondrial uncoupling proteins and resistance to cell hypertrophy and cell death. Evidence suggests that caspase-2 deficiency protects mice from diet-induced obesity, metabolic syndrome, and non-alcoholic fatty liver disease, which has led to speculation that caspase-2 activity in NASH is beyond apoptosis. However, it is important to note that NASH development is associated with other types of cell death, such as necroptosis and ferroptosis [128, 129].

Caspase-8 activation has been detected in liver biopsies from patients with alcoholic liver disease (ALD). In addition, researchers noted caspase-8 activation in the liver of wt mice after chronic ethanol feeding for 8 weeks (about 2 months) compared with hepatocyte-specific caspase-8 knockout mice (Casp8^Δhepa^). Ethanol-fed Casp8^Δhepa^ mice still show alcohol-induced liver damage; however, they present attenuated steatosis and reduced hepatic triglyceride and free fatty acids. These findings indicate that caspase-8 activation is a critical event in alcohol-dependent fat metabolism [130]. Taken together, activation of at least caspase-2, caspase-3, and caspase-8 has a pivotal role in liver injury in a cell death-independent manner.

In stroke, caspases are important players in the inflammatory, apoptotic, and vascular processes that dominate cerebral ischemia. However, there is increasing evidence for several non-apoptotic functions of caspases in the post-ischemic brain. Caspase-1 contributes to inflammation in ischemic stroke by processing the pro-inflammatory cytokine IL-1β into its active form. In addition, caspase-1 is upregulated in acute stroke, mainly mediating pyroptosis, and compromising blood-brain barrier integrity via lytic cellular death and inflammatory cytokine release [131]. Caspase-11 knockout mice are protected from stroke, suggesting the importance of its activation in the stroke. Caspase-11 is one of several upstream caspases in the cascade that mediates the activation of both caspase-1 and caspase-3, which can result in cytokine release and/or apoptosis, respectively, leading also to stroke induction [132]. Thus, this enzyme might be linked bidirectionally to stroke. Although genetic knockout of caspase-1, caspase-3, and caspase-6 provides neuroprotection against stroke [133], one may ask whether apoptosis plays a critical role in ischemic stroke. Researchers observed more cleaved caspase-3 after permanent middle cerebral artery occlusion in rats. They noted that cleaved caspase-3 co-stained with markers directed against astrocytes and macrophages/microglia, suggesting that the level of this protein is associated with the cellular responses to stroke such as reactive astrogliosis and the infiltration of macrophages independent of apoptosis [134]. In addition, there is increased cleaved caspase-3 in neuronal precursor cells (NPCs) in the subventricular zone (SVZ) during the period of stroke recovery of ischemic mice, with no evidence of apoptosis. Moreover, caspase-3 negatively regulates the proliferation of NPCs by reducing the phosphorylation of Akt. Inhibition of its activity significantly promotes the proliferation and migration of SVZ NPCs and results in a substantial increase in neuronal regeneration and functional recovery after stroke [135].

As mentioned above, several caspases are activated in hair follicles and the surrounding skin. Caspase-14 is involved in keratinocyte differentiation leading to normal skin cornification. Remarkably, by analyzing biopsies from psoriatic skin plaques of patients with psoriasis, researchers found that a disturbed keratinization process in the parakeratotic region of psoriatic skin is associated with a very low level of caspase-14 in the cytoplasm and absence in the nucleus, suggesting that the deregulation of caspase-14 synthesis is related to disturbance of the normal, terminal keratinocyte differentiation program in patients with psoriasis [68, 136]. However, caspase-14 is probably not the cause of the development of parakeratotic plaques, as caspase-14-deficient mice do not show spontaneous parakeratosis [69]. Treating the parakeratotic plaques of patients with a vitamin D3 analog upregulates caspase-14 and coincides with the amelioration of the lesions. Likewise, in the flaky skin (fsn/fsn) mouse model of psoriasis, topical epigallocatechin gallate treatment upregulates caspase-14 and ameliorates psoriasis. Therefore, it is more likely that caspase-14 downregulation results from the impairment of terminal differentiation or upregulation of transcriptional repressors. The absence of caspase-14 in psoriatic plaques may lead to the formation of a defective barrier and, therefore, to the aggravation of psoriatic lesions [69, 137]. Regarding the involvement of other caspases deregulation in skin disease, caspase-1 is upregulated in androgenic alopecia, while caspase-8 deficiency in keratinocytes triggers chronic skin inflammation in mice [138]. Overall, caspase deregulation plays an essential role in disease progression, and, in these cases, caspases might represent a promising therapeutic target.

## Therapeutic implications of caspases in diseases

### Caspase inhibitors

**Fig. 5:**
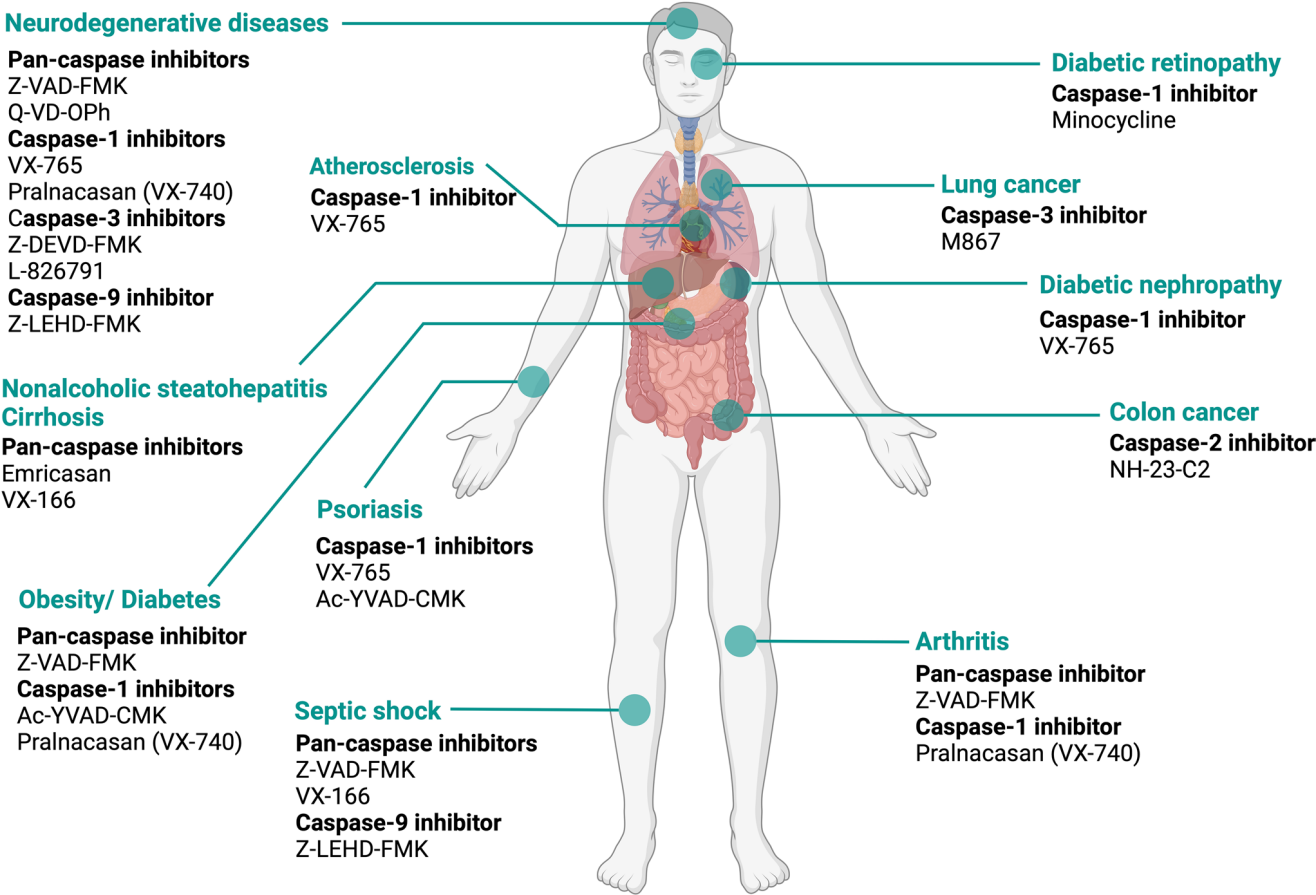
Therapeutic implications of caspase inhibitors

Several caspase inhibitors have been developed as a potential therapeutic intervention for numerous disorders including inflammatory diseases, neurodegenerative diseases, liver disease, and cancer. They have been reviewed in detail previously [139] and are summarized in (Table 2; Fig. 5). Briefly, at present, based on the origin caspase inhibitors can be divided into two categories. Natural caspase inhibitors, such as CrmA [140], p35 family [141], and IAPs [142], belong to the first category. The second one includes synthetic caspase inhibitors which are classified into peptide and non-peptide compounds, as well as the allosteric caspase inhibitors. As an example of peptide inhibitors, one can mention Ac-YVAD-CHO, a potent caspase-1 inhibitor. Unfortunately, its therapeutic activity is limited by poor membrane permeability and instability. On the other hand, pralnacasan (VX-740), a non-peptide caspase-1 inhibitor is orally active [143]. Pralnacasan was used for the treatment of RA and osteoarthritis (OA) [144]. Yet, high doses of pralnacasan in animal models induced liver toxicity [145]. The allosteric caspase inhibitors target the allosteric site of caspases, thus allowing the binding of the inhibitor to the dimer interface of the enzyme. Though, there is no allosteric caspase inhibitor progressed to the clinical trial until now [139]. Several caspase inhibitors are patented for therapeutic use. In brief, peptide caspase-1 inhibitors such as z-WEHD-FMK and Ac-YVAD are used for the treatment of dermatitis. On the other hand, non-peptide caspase inhibitors, such as MJL-001i, MJL-002i, and MJL-003i, are used for treatment and/or prophylaxis of HD by blocking the cleavage of Htt mediated by caspase-3 or caspase-6. z-DEVD-fmk, a caspase-3 inhibitor, promotes the proliferation of nerve cells in the injured brain of the rat’s post-stroke. For more details, caspase inhibitors are reviewed in the following articles [146, 147].

**Table 2:** Caspase inhibitors in preclinical and clinical models of various diseases

Caspase inhibitor	Disease/ disorder	Preclinical/Clinical use/ trial	Ref.
1- Pan- caspase inhibitor			
Z-VAD-FMK	Arthrities, septic shock, ALS, diabetes	Pre-clinical (in- vivo)	[148–150]
Emricasan	Cancer	Pre-clinical (in vitro)	[151]
VX-166	NASH cirrhosis	Phase II (failed)	[152]
Q-VD-OPh	NASH cirrhosis	Phase II	[153]
	Septic shock	Pre-clinical (in- vivo)	[154]
	CNS injuries, AD, HD, PD, AIDS	Pre-clinical (in- vivo)	[155–157]
2- Caspase- 1 inhibitor			
VX-765	Psoriasis, epilepsy	Completed Phase II, Phase IIa	[158, 159]
	AD, diabetic nephropathy, atherosclerosis	Pre-clinical (in- vivo)	[117]
Ac-YVAD-CMK	Psoriasis, obesity	Pre-clinical (in- vivo)	[160, 161]
Pralnacasan (VX-740)	Arthritis	Phase II	[144]
	Epilepsy, obesity	Pre-clinical (in- vivo)	[162, 163]
3- Caspase- 2 inhibitor			
NH-23-C2	Colon cancer	Pre-clinical (in-vitro)	[164]
4- Caspase- 3 inhibitor			
M867	Lung cancer	Pre-clinical (in-vitro)	[165]
Z-DEVD-FMK	CNS injuries, AD	Pre-clinical (in-vitro)	[166, 167]
L-826791	CNS injuries	Pre-clinical (in-vitro)	[168]
5- Caspase- 9 inhibitor			
Z-LEHD-FMK	CNS injuries, Septic shock	Pre-clinical (in- vivo)	[169]

### Caspase activators

Given that caspase activation induces cellular demise and differentiation, it can be utilized as a therapeutic strategy for specific diseases, including cancer. One of the promising caspase inducer therapeutic strategies is inducible caspase-9 (iCasp9), a suicide gene that can be activated to cause PCD [170]. iCasp9 consists of a fusion between human caspase-9 and an inducer-binding domain. This fusion can be combined in a dimeric form by a chemical inducer of dimerization (CID), AP1903, or AP20187. Alternatively, iCasp9 can be activated by rapamycin in a short dosing regimen. The advantage of using rapamycin would be its market availability and good biodistribution [171]. The iCasp9 protein is dimerized and activated through induced proximity of the inducer-binding domain, resulting in activation of the apoptotic pathway. iCasp9 has been used to induce cell death in cancer therapies as well as adoptive cell therapies (Table 3). In clinical trials, modified T cells that can recognize tumor-associated antigens have demonstrated efficacy against cancerous cells and have resulted in remarkable clinical outcomes. Nevertheless, antigen-specific T cells can exhibit severe, and potentially lethal, toxicities including graft-versus-host disease (GVHD) because of inadequate regulation of their activation, expansion, and persistence. Therefore, the development of a safety switch is a critical step. Ideally, this switch should be non-immunogenic and activated by an inert agent. iCasp9 preclinical and clinical trials have shown that iCasp9 can be used successfully as a safety switch with T-cell therapy. The expression of iCasp9 in human T cells does not impair their phenotypic, functional, or antigenic specificity. A single administration of AP1903 (10 nM), also known as an inert CID, triggers apoptosis in more than 90% of iCasp9-transduced cells rapidly within 2 h. Hence, the incorporation of the iCasp9 safety switch provides the ability to eliminate these modified T cells in the event of unexpected GVHD and improves the safety of T cell therapy [172, 173]. FGFR4–CAR–iCasp9 model has specifically and effectively targeted rhabdomyosarcoma (RMS) cells expressing high levels of FGFR4. Co-culturing FGFR4–CAR–iCasp9 T cells with human RMS cell line (RH4) reduced the killing effect of CAR-T cells and the release of TNF-α, IFN-γ, IL-2, and IL-6 compared to the control group [174]. Likewise, induced pluripotent stem cells (iPSCs) hold significant potential for the field of regenerative medicine. Human iPSCs harboring the iCasp9 suicide safety switch undergo PCD in over 98% of iPSCs when exposed to AP20187 (0.1 nM) [175]. Comparably, mesenchymal stromal cells (MSCs) that express iCasp9 are selectively eliminated after AP1903 treatment either in vitro or in vivo in a murine model [176].

**Table 3:** Clinical trials that have used the iCasp9 system

Study title	Biological intervention	Drug intervention	Disease	Phase	Study Status	Date of completion	Ref
CASPALLO: Allodepleted T Cells Transduced with Inducible Caspase 9 Suicide Gene	Allodepleted T Cells		AML CML NHL MDSs	Phase1	Active not recruiting	2026-07	[183]
Anti-CD19 CAR-T Cells with Inducible Caspase 9 Safety Switch for B-cell Lymphoma	iC9-CAR19 T cells	Bendamustine Fludarabine AP1903 Cyclophosphamide	Lymphoma B cell lymphoma Lymphatic disease Immune System Diseases Immunoproliferative Disorders	Phase1	Recruiting	2043/03	[184]
Compassionate use of car t cells targeting the cd19 antigen and containing the inducible caspase 9 safety switch	iC9-CAR19 cells	AP1903 Cyclophosphamide Fludarabine	ALL Immune System Diseases Immunoproliferative Disorders		No longer available		[185]
T Lymphocytes (LT) Expressing iCASP9 and ΔCD19 in Allogeneic Haematopoietic Transplantation.	T lymphocytes iCASP9 ΔCD19	AP1903	GVHD Hematological Malignancies	Phase1/ Phase2	Unknown	2021-12	[186]
Study of Autologous CAR-T Cells Targeting B7-H3 in TNBC iC9-CAR.B7-H3 T Cells	iC9-CAR.B7-H3 T Cell Therapy	Cyclophosphamide Fludarabine	Breast Cancer TNBC Relapse/Resistant Cancer	Phase1	Not recruiting yet	2028-05	[187]
Autologous CAR-T Cells Targeting B7H3 in Ovarian Cancer iC9-CAR.B7-H3 T Cells	iC9-CAR.B7-H3 T cells	Cyclophosphamide Fludarabine	Ovary Neoplasm Ovarian Cancer/ Recurrent	Phase1	Not recruiting yet	2026-03	[188]
Study of CAR T-Cells Targeting the GD2 With IL-15 + iCaspase9 for Relapsed/Refractory Neuroblastoma or Relapsed/Refractory Osteosarcoma	iC9.GD2.CAR.IL-15 T-cells	Cyclophosphamide Fludarabine	Neuroblastoma Osteosarcoma	Phase1	Recruiting	2039/6	[189]

iCasp9 also induces cell death in various cancer cell lines: HCT116 (colorectal cancer) [177], H1299 (NSCLC) [178], and MCF-7 (breast cancer) [179]. Dimerization of iCasp9 with AP20187 (10 nM) in HCT116 cells significantly increases apoptosis within 24 h, with elevated cleavage of caspase-3 and PARP measurable at 1 h after AP20187 administration. Additionally, in a tumor mouse model, subcutaneous injection of immunodeficient mice with iCasp9 transfected colorectal cancer stem cells along with treatment with intraperitoneal treatment of AP20187 for 15 days results in a reduction in tumor size compared with the untreated control. Nevertheless, following this initial reduction, tumors continue to grow. The regrowth of the tumor could be due to an escape from therapy due to the availability of the dimerizer, epigenetic silencing of the iCasp9 construct by promotor hypermethylation [173], or in vivo resistance [177]. Similarly, 24 h after treatment with iCasp9-transduced H1299 NSCLC cells with AP20187, more than 80% of the cells are apoptotic compared with non-transduced cells. In the SCID-Beige NSCLC xenograft mouse model inoculated with A549 cells, iCasp9-transduced infusion followed by AP20187 treatment reduces tumor growth [178]. Moreover, iCasp9 transfection induces apoptosis by more than 60% in MCF-7 breast cancer cells compared with the control after 48 h of incubation with the CID (AP1903) [179].

To summarize, iCasp9 dimerization triggers rapid and highly effective apoptosis in cancer cells. Thus, this construct serves as a compelling tool in the management of cancer. Overall iCasp9 is considered an excellent candidate as a safety switch because it is a nonimmunogenic, bioinert, rapid, and effective activator, and it does not interfere with other drugs. However, the clinical concerns about its in vivo resistance, and the possibility of induction of cytokine-release syndrome (CRS) are still unclear and further studies are required. In a phase 1 trial to evaluate the effectiveness of iCasp9 T cells in adult patients who are receiving haploidentical stem cell transplantation for high-risk hematologic malignancies, one of the three patients experienced donor-derived Epstein–Barr virus–associated post-transplant lymphoproliferative disorder (EBV-PTLD), followed by a significant increase in iCasp9 T cells and CRS. This indicates that iCasp9 T cells retain a high in vivo proliferative capacity and function and could cause CRS in response to *de novo* lymphoma development [180].

Because caspases are involved in the differentiation process, using iCasp9 could be beneficial to treat neuroblastoma, one of the most common solid tumors in children. Neuroblastoma cells can differentiate into several types of nerve cells and exhibit a high level of multipotency. Neural differentiation inhibits cell and tumor growth, thus preventing the progression of neuroblastoma and facilitating its treatment. Human neuroblastoma SH-SY5Y cells transfected with iCasp9 and followed by AP1903 treatment change from epithelial-like cells to more branched neuronal (star-shaped) differentiated cells, the morphological character of astrocytes. Furthermore, caspase-9 activation in SH-SY5Y cells can lead to G_1_-phase cell cycle arrest, perhaps due to cleavage of non-apoptotic proteins that are involved in the G_1_/S transition. A major concern is the high dose of the dimerizer (50 nM AP1903) used to induce differentiation by activated iCasp9: Could it result in apoptosis of SH-SY5Y cells? However, researchers found this dose of AP1903 results in the highest degree of differentiation of SH-SY5Y cells without causing apoptosis. There may be variable cell sensitivity to caspase-9 activity, causing resistance to apoptosis at higher inducer dosages. Taken together, the iCasp9/AP1903 system is a promising candidate as a novel strategy to trigger differentiation as part of neuroblastoma therapy [181].

## Conclusion

Caspases and their apoptotic activity in cells have been the subject of numerous studies and reviews. Nevertheless, the overwhelming evidence suggests that caspases have a broader range of functions than previously anticipated. Caspases are crucial for regulating cell death, myocardial development, neurogenesis, osteogenesis, and skin cornification under physiological conditions. Under pathological conditions, caspases play an indispensable role in lymphocyte recruitment and wound healing. However, we still do not fully understand the roles of each caspase and their interconnections. For example, we do not know why some living organisms, such as humans or mice, perish without some caspases, while others, like plants, thrive. Moreover, it is unclear whether caspase-14 is the only caspase that contributes to the formation of the skin barrier, whether caspase-2 influences the cognitive behavior of mice or humans, whether caspase-2 or other caspases play a role in the aging process, and why some caspases are unique to certain living organisms. We do know that overexpression of caspase-1, caspase-2, caspase-3, caspase-6, and caspase-8 is linked to Aβ accumulation and cognitive decline in mouse models and patients with neurodegenerative diseases. Furthermore, deregulated activation of caspase-2, caspase-3, and caspase-8 contributes to the exacerbation of liver disease injury, while caspase-1, caspase-3, and caspase-6 knockout protect against stroke. Indeed, caspase-targeted interventions have therapeutic potential for several diseases, such as AD, psoriasis, NASH cirrhosis, septic shock, and cancer. To date, however, the side effects and risks of caspase inhibitors or activators have outweighed their benefits. Additional studies are required to deeply understand the functions of caspases. Even with over half of a century of caspase studies, the light at the end of the tunnel has yet to appear.

## Data Availability

Not applicable.
